# Echographie et tomodensitométrie dans les appendicites retro caecales

**DOI:** 10.11604/pamj.2013.14.117.2169

**Published:** 2013-03-27

**Authors:** Amadou Abdoulatif, N'timon Bidamin, Marouane Ahmed, Bichri Mustapha

**Affiliations:** 1 Service de radiologie du Centre Hospitalier de l'Arrondissement de Montreuil dur Mer (CHAM), France; 2 Service de radiologie Centre Hospitalier de Douai, France

**Keywords:** Appendicite retrocaecale, tomodensitométrie, échographie, retrocaecal appendicitis, CT scan, ultrasound

## Abstract

Le but de cette étude était de déterminer entre l'échographie et la tomodensitométrie, le moyen d'imagerie le plus approprié dans le diagnostic des appendicites retrocoecales. Il s'agissait d'une étude rétrospective ayant concerné les dossiers des patients qui ont bénéficié d'une échographie et d'une tomodensitométrie pour un syndrome appendiculaire. Nous avons retenu les dossiers des patients chez lesquels le diagnostic d'appendicite aigu a été posé par l'un des deux moyens d'imagerie, et confirmé par l'intervention chirurgicale. pendant 1 an, 19 cas d'appendicites retroceacales aiguës ont été retrouvées. L'âge moyen des patients était de 36 ans, avec une prédominance masculine. Les manifestations cliniques étaient dominées par la douleur dans l'hémi abdomen droit. La fièvre était présente dans tous les cas. On notait également une hyperleucocytose, et une élévation de la CRP chez tous les patients. L'échographie était normale dans 6 cas (32%), alors qu'elle mettait en évidence une infiltration de la paroi caecale, avec infiltration de la graisse péri caecale dans 13 cas (68%). La TDM abdominopelvienne a mis en évidence dans tous les cas, un appendice retrocaecale, épaissi avec un diamètre supérieur à 7 mm, et une infiltration de la graisse péri appendiculaire. La chirurgie avait confirmé le diagnostic d'appendicite retrocaecale. Les suites opératoires étaient satisfaisantes, sans complications. Le diagnostic d'une appendicite retrocaecale n'est pas aisé. L'échographie est le plus souvent non concluente. La TDM apparait comme le moyen d'imagerie de choix dans le diagnostic des appendicites retrocaecales.

## Introduction

L'appendicite aigue représente la première urgence chirurgicale digestive en pratique quotidienne. Cependant, cette affection aussi banale soit elle, reste de diagnostic difficile source de controverses entre les radiologues et les cliniciens [1]. Ceci conduit à un retard diagnostique, responsable de complication à type de perforation appendiculaire qui survient chez près de 30% des patients et augmente la morbidité du traitement chirurgical [2]. Les difficultés diagnostiques seraient liées dans certaines situations à la position retrocaecale de l'appendice [3]. Le groupe de travail de l'ANDEM, à partir des travaux de FLAMMAND [4], a établi des signes cliniques permettant d'éviter les appendicectomies blanches. Malheureusement, l'appendicite retrocoecale est de manifestations cliniques atypiques [5]. Les limites de la clinique emmènent le plus souvent à recourir à l'imagerie. L'échographie et la TDM sont les moyens d'imagerie le plus souvent utilisés. Leur sensibilité dans le diagnostic des appendicites en général serait proche avec un avantage pour la TDM [6]. Mais rares sont les études qui rapportent leur sensibilité dans les cas particuliers d'appendicites retrocoecale. Il nous a semblé nécéssaire d'effectuer une analyse retrospective des appendicites retrocoecales confirmées chirurgicalement, afin de déterminer entre l'échographie et le scanner, le moyen d'imagerie ayant permis de faire le diagnostic de certitude.

## Méthodes

Notre étude a été rétrospective. A partir des bases de données de services de radiologie et de la chirurgie, nous avons sélectionné de façon rétrospective, les dossiers des patients admis pour syndrome appendiculaire, chez lesquels le diagnostic d'appendicite retrocaecale a été formellement posé par la chirurgie et qui ont bénéficié d'une échographie et d'un scanner abdominopelvien. Nous avons revu pour chaque dossier, les comptes rendus de ces deux moyens d'imagerie ainsi que les comptes rendus opératoire. Nous avons L'échographe utilisé est de marque SIEMENS. Les sondes sont de type multifréquence, avec des fréquences variant de 9MHZ à 18MHZ. Le critère échographique admis dans le diagnostic d′appendicite retrocaecale dans notre étude est la visualisation d′un appendice non compressible, apéristaltique, de plus de 6 mm de diamètre transverse [7], situé en arrière du caecum, associé ou non à des signes indirects à savoir l'épaississement de la paroi coecale de plus de 5mm, l'aspect hyperéchogène de graisse péritonéale, et une collection liquidienne pelvienne. L'appareil TDM utilisé est mltibarrette (40 barrettes), de marque PHILIPS. La technique TDM a consisté à une acquisition volumique sans injection et avec injection du contraste au temps portal. Le diagnostic tomodensitométrique est fait sur la visualisation d'un appendice épaissi mesurant au moins 7mm de diamètre [8], situé en arrière du caecum, associée ou non à des signes indirects à savoir l'épaississement de la paroi coecale, l'aspect hyperdense de la graisse péritonéale et la collection liquidienne intrapéritonéale. Nous avions recueilli pour chaque patient, les données épidémiologiques, cliniques, biologiques.

## Résultats

**Figure 1:**
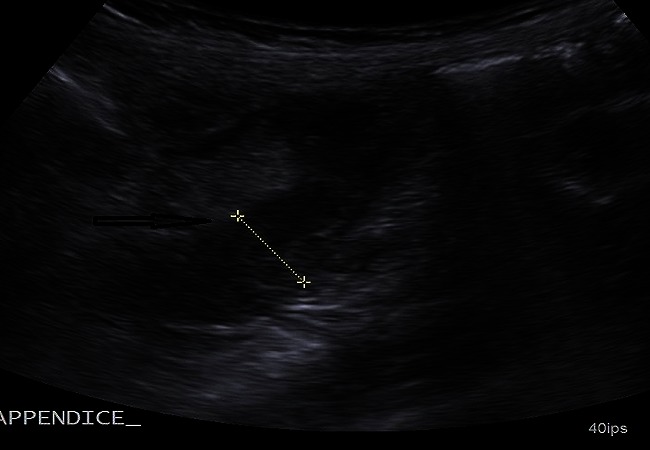
Echographie en coupe longitudinale montrant un appendice épaissi (flèche horizontale), mesurant 12mm de diamètre, en arrière du caecum, avec une infiltration de la graisse péri appendiculaire d'aspect hyperéchogène

**Figure 2:**
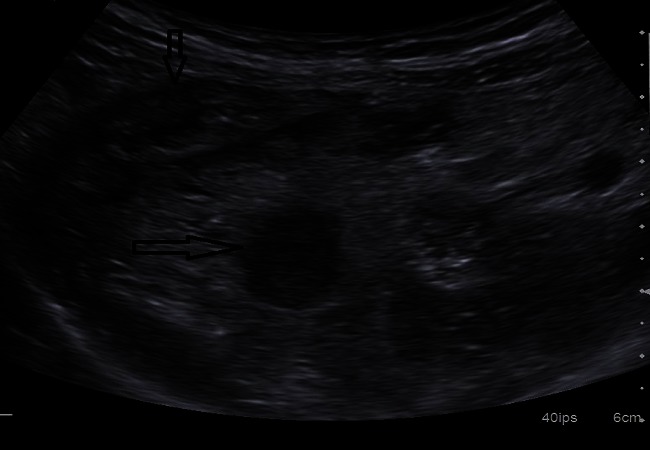
Echographie en coupe longitudinale montrant un appendice épaissi (flèche horizontale), mesurant 12mm de diamètre, en arrière du caecum (flèche verticale), avec une infiltration de la graisse péri appendiculaire d'aspect hyperéchogène

**Figure 3:**
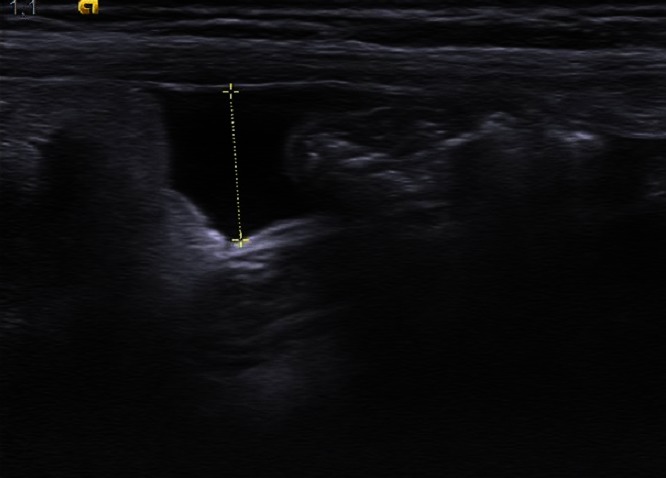
Epanchement liquidien dans la fosse iliaque droite lié à une appendicite

**Figure 4:**
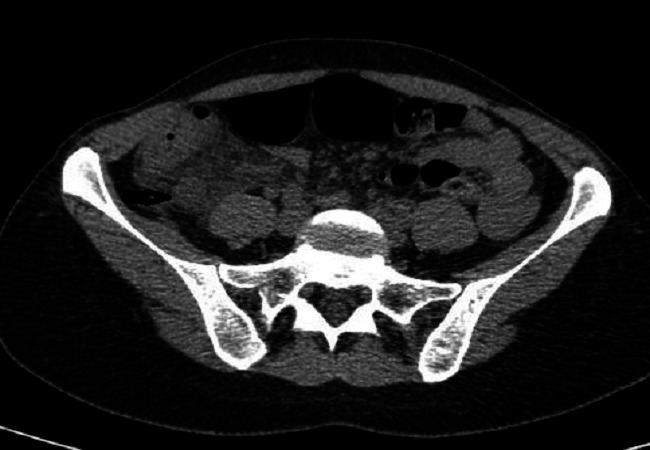
TDM abdomino-pelvienne coupe axiale, montrant en un appendice épaissi (flèche horizontale), en arrière du caecum, avec une infiltration de la graisse périappendiculaire

**Figure 5:**
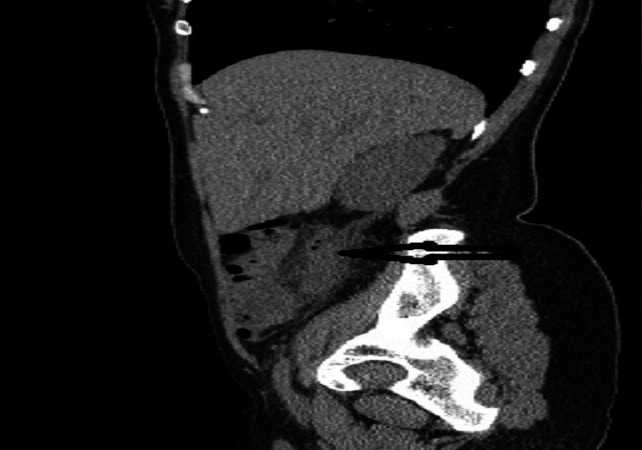
TDM abdomino-pelvienne en coupe sagittale montrant un appendice épaissi (flèche horizontale), mesurant 12mm de diamètre, en arrière du caecum, avec une infiltration de la graisse péri appendiculaire

Parmi 193 cas d'appendicite aigue opérée en 12 mois, 23 cas, soit 12% étaient retrocaecales dont l'âge moyen était de 36 ans (écart allant de 24 ans à 63 ans), avec un sex-ratio de 14 hommes sur 9 femmes. Le maitre symptôme était une douleur de la fosse iliaque droite. Elle était présente dans tous les cas. L'irradiait dans le flanc droit et dans la fosse lombaire droite était noté dans 11 cas (48%), uniquement dans le flanc droit dans 9 cas (39%), et uniquement dans la fosse lombaire dans 3 cas (13%). La fièvre était présente dans 17 cas (74%). Il était noté une hyperleucocytose, et une élévation de la CRP chez tous les patients. L'échographie réalisée dans ce contexte clinique, n'a permis de visualiser le signe direct d'appendicite retrocoecale que dans 2 cas (8,7%) (Tableau 1, Figure 1, Figure 2). Elle avait mis en évidence dans 9 cas (39%) des signes indirects (Tableau 2, Figure 3), soit de façon associée ou soit de façon isolée. Le principal signe indirect échographique était représenté par l'infiltration de la graisse péritonéale (Tableau 3). La TDM abdominopelvienne complémentaire réalisée avait été réalisée. Elle a montré dans tous les cas le signe direct d'appendicite en situation retro caecale (Tableau 1, Figure 4, Figure 5). Elle a décelé des signes indirects associés ou isolés dans les cas (Tableau 2). L'infiltration de la graisse péritonéale péri appendiculaire était le signe indirects TDM le plus présent. La répartition des signes indirects TDM est consignée dans le Tableau 3.

**Tableau 1:** Comparaison de la visualisation de l'appendicite retrocaecale entre l'échographie et la TDM

Résultat	Echographie (n= 23)	TDM (n = 23)
**Positif**	2 (8,7%)	23 (100%)
**Négatif**	21 (91,3%)	0 (0%)

X2= 41,85, P inf à 0,05 différence très significative ; La TDM est plus sensible que l'échographie dans le diagnostic de l'appendicite retrocaecale

**Tableau 2:** Comparaison de la présence de signe indirect entre l'échographie et la TDM

	Echographie (n= 23)	TDM (n = 23)
**Positif**	9 (39,13%)	23 (100%)
**Négatif**	14 (60, 87%)	0 (0%)

X2 = 20,12 P inf à 0,05 Différence significative. La TDM est plus sensible que l'échographie dans le diagnostic des signes indirects dans les appendices retrocaecales

**Tableau 3:** Comparaison de la présence chaque type de signe indirect entre l'échographie et la TDM

	Echographie (n= 23)	TDM (n = 23)
Epaississement de la paroi coecale	5 (21,7%)	12 (52,17%)
Infiltration de la graisse péritonéale	9 (39,13%)	23 (100%)
Collection liquidienne pelvienne	4 (17,4%)	7 (30,43%)

X2= 0,566 ; P sup à 0,05 différence non significative. Pour chaque signe indirect pris isolément, dans les appendicites retrocaecales, l'échographie a une sensibilité équivalente à la TDM

L'analyse statistique réalisée a montré une différence très significative entre l'échographie et la TDM dans le diagnostic de l'appendicite retrocoecale par le signe direct. Il existe également une différence significative entre les deux moyens d'imagerie dans la détection des signes indirects. Par contre, pour chaque signe indirect isolé, il n'existe pas de différence significative entre l'échographie et la TDM. Ainsi la sensibilité de la TDM apparait supérieure que celle de l'échographie dans le diagnostic de l'appendicite retrocoecale.

La chirurgie réalisée chez tous les patients, avait permis de faire une résection de l'appendicite retrocoecale. Les suites opératoires étaient satisfaisantes, sans complications.

## Discussion

L'appendicite ectopique, le plus souvent retrocaecale représente un problème diagnostic. De par son siège, elle est de manifestation clinique souvent atypique, à l'origine des erreurs ou de retard diagnostic [9]. Dans cette difficulté diagnostique, le recours à l'imagerie est indispensable pour la prise d'une décision chirurgicale. Si l'échographie demeure l'examen de première intention dans le syndrome appendiculaire, sa sensibilité ne serait pas prouvée dans les appendicites retrocaecales. En effet, rares sont des études dans la littérature qui rapportent le diagnostic des appendicites retro caecales à l'échographie. Notre étude a retrouvé performance échographique faible dans la visualisation de l'appendicite retrocoecale. Dans l'étude de Kouamé et al [3], 93% des difficultés diagnostiques échographique de l'appendicite étaient liées à sa localisation retro-caecale. Selon Taourel et al [10], les faux négatifs en échographie sont rencontrés dans les appendicites ectopiques. L'absence de diagnostic échographique direct de l'appendice a amené certains auteurs à faire appel aux signes indirects. Ces signes indirects ne sont pas spécifiques d'une appendicite, et pourraient être responsable d'appendicectomie blanche. En effet certaines pathologies telles qu'une colite droite, une diverticulite, ou une tumeur caecale ou la maladie de chron peuvent présenter les mêmes signes indirects. Dans certains cas, l'apparition des signes indirects à l'échographie est l'expression de la survenue d'une complication telle un abcès appendiculaire ou une péritonite appendiculaire. L'étude de Kouamé et al [3], basé sur les signes échographiques a rapporté de nombreuses complications. Dans notre étude, nous n'avons pas retrouvé de complication. Pour éviter ces complications, après une échographie non concluante devant un syndrome appendiculaire, certains auteurs [10, 11, 12, 13], recommandent le recourt immédiat à la TDM. Notre étude a montré une nette supériorité de la sensibilité de la TDM sur l'échographie dans les appendicites retrocoecales. Elle a permis le diagnostic direct dans tous les cas, permettant une prise en charge chirurgicale rapide. Les cas d'appendicite retrocoecale rapporté dans la littérature [9, 10, 14] sont de diagnostic TDM. Selon Taourel [10], l'appendicite retrocaecale représente une indication de la TDM.

## Conclusion

De par sa position atypique, et ses manifestations cliniques inhabituelles, l'appendicite retrocaecale pose un problème diagnostique. Le recourt à l'imagerie est indispensable. La TDM apparait plus sensible que l'échographie dans le diagnostic et la prise en charge de cette affection.
